# Effect of Obstetric Fine Nursing on Pain during Natural Childbirth and Postpartum Recovery

**Published:** 2018-11

**Authors:** Youliang OU, Yanli ZHOU, Ping XIANG

**Affiliations:** 1. Dept. of Obstetrics and Gynecology, Nanfang Hospital of Southern Medical University, Guangzhou, China; 2. Dept. of Medical Quality Management, Nanfang Hospital of Southern Medical University, Guangzhou, China

**Keywords:** Fine care, Natural childbirth, Pain, Labor, Postpartum recovery

## Abstract

**Background:**

This study aimed to observe the effect of fine nursing on pain and postpartum recovery during natural delivery of pregnant women.

**Methods:**

The clinical data of 192 primiparas who were expecting labor in Nanfang Hospital, Southern Medical University, Guangzhou, China from 2015–2016 were retrospectively analyzed. Among them, 100 cases were treated with fine nursing before and after delivery, and 92 cases were treated with routine nursing. The pain rate, the second stage of labor, the amount of bleeding 2 hours postpartum, and postpartum recovery were compared between the two groups.

**Results:**

The number of cases of grade 0 and 1 pain and the good rate of pain were significantly higher in the observation group than in the control group, the number of cases with grade 2 pain in the observation group was significantly lower than that of the control group (*P*<0.01). The second stage of labor and the 2h postpartum hemorrhage in the observation group were significantly lower than those in the control group (*P*<0.001). The number of maternal cases with good postpartum sleep, good lactation and good mental health in the observation group was significantly higher than that in the control group (*P*<0.05).

**Conclusion:**

Fine nursing before and after delivery can reduce maternal pain rate, shorten the second stage of labor, reduce the amount of bleeding after 2 hours postpartum, promote good sleep, lactation, psychological conditions, reduce postpartum infection rate, which is conducive to maternal body recovery, worthy of clinical promotion.

## Introduction

Childbirth is a natural physiological phenomenon of women, but it is also a strong source of stress because it affects women’s own physical and psychological conditions, and even family functions (1, 2). Therefore, ensuring maternal and physical health has always been an important concern in the field of public health (3). In general, due to lack of understanding or experience of the childbirth process, primipara are more likely to have fear of childbirth than multipara, which causes anxiety during pregnancy (4), so it is of great significance for the obstetrics to adopt appropriate nursing mode to relieve primipara pain and promote good labor and postpartum recovery. However, with the development and progress of the medical model, the application of fine nursing mode before and after childbirth in clinical nursing work is also increasing (5).

Maintaining a good relationship between the mother and the daily caregivers can reduce the pain caused by contractions and promote the postpartum recovery of the mother, making maternity more positive in life (6). Fine care is an emerging pattern which requires nurses not only to strengthen the attention of patients, but also to pay attention to the factors affecting the rehabilitation of diseases, such as the patient’s psychological condition, environment, physical factors, etc. (7, 8). Fine care is a personalized approach to care based on the patient’s social, cultural, and physical and mental needs. It includes guidance and interventions on diet, psychology, behavior, post-operative hygiene, post-operative pain, and breastfeeding (9), which is conducive to improve the surrounding environment of maternity, reduce or even eliminate the fear and anxiety caused by approaching childbirth (10).

We aimed to study the effect of pre-delivery fine nursing on the pain rate of pregnant women during natural childbirth and the postpartum recovery of maternal, and provided a theoretical basis for the choice of clinical nursing work mode.

## Materials and Methods

### General Information

The clinical data of 192 primiparas who were expecting labor in Nanfang Hospital, China from June 2015 to June 2016 were retrospectively analyzed. The average age was (26.85±5.67) years old, and the observation group was treated with fine nursing before delivery, 100 cases in total. A total of 92 cases in the control group were treated with routine care. There were no significant differences between the two groups in the general data of maternal (*P*>0.05). All women were primipara, no history of drug allergy, normal liver and kidney function, all excluded pregnancy complications, cesarean section indications, multipara (6).

The study was approved by the Medical Ethics Committee of Nanfang Hospital, Southern Medical University, and the maternity and family members gave informed consent (Table 1).

**Table 1:** General information [n(%)]

***Index***		***Observation (n=100)***	***Control (n=92)***	***t/χ^2^***	***P***
Age (yr)				0.062	0.875
	≤30	69(69.00)	65(70.65)		
	>30	31(31.00)	27(29.35)		
Height (cm)		161.78±5.77	160.84±3.98	1.303	0.194
Weight (kg)		64.85±4.37	63.89±3.27	1.712	0.089
Gestational week (week)		37.75±2.86	38.21±1.72	1.336	0.183
Ischial spine diameter (cm)		9.58±1.58	9.23±1.19	1.722	0.087
Fetal biparietal diameter (cm)		8.12±1.37	8.01±1.29	0.572	0.568
Obstetric conjugate (cm)		11.37±2.08	11.65±1.76	1.003	0.317
Conjugate of outlet (cm)		9.28±1.83	9.11±1.62	0.679	0.498
Educational level				1.500	0.244
	Below college	38 (38.00)	43 (46.74)		
	College or above	62 (62.00)	49 (53.26)		

### Nursing methods

The control group received routine obstetric care, including diet guidance, life care, medication, health education, and psychological care.

The observation group received obstetric refinement care. Psychologically, the nursing staff establishes and maintains a good relationship with the mother, and helps the primipara to reduce the fear, pessimism and anxiety of the natural childbirth through communication and encouragement. At the same time, the staff explains the delivery process and the childbirth breathing guidance, and the childbirth precautions for the mother to make the mother fully psychologically prepared before giving birth; secondly, in terms of diet, the mother can eat more high-protein, high-fiber food with more diversify and increase energy intake; in terms of behavior, before childbirth, under the guidance of a doctor, exercise properly in a comfortable position, and get out of bed as soon as possible after childbirth; in terms of postoperative pain, urinate as early as possible, keep the wound clean and prevent infection; in terms of postoperative hygiene, due to the large physical exertion during the childbirth and the sweating, the staff should try to keep the body dry and clean to prevent getting cold. Hygiene products and clothes are changed frequently to keep the vulva clean; in terms of breastfeeding, to explain the advantages of breastfeeding for the mother, breastfeed as soon as possible, stimulate the breast to secrete milk as soon as possible, and encourage the mother to face breastfeeding with an optimistic attitude. Nipple and breast care: massage, hot compress to prevent breast pain and mastitis, moisturize the nipple with milk or nipple cream to prevent nipple splitting caused by baby sucking. Postpartum fine care includes measuring blood pressure, compression of the fundus to promote drainage, observation of vaginal bleeding volume and color, early contact between newborn and maternal (11).

### Observation indicators

The pain rate, the second stage of labor, the 2h postpartum hemorrhage, and postpartum recovery were compared between the two groups.

The degree of pain was divided into four levels, which were 0, 1, 2, and 3, respectively. The higher the level, the greater the degree of pain will be.

Good rate of pain = (number of cases of 0+1) / total number of cases ×  100%

Weighing method combined with volumetric method was used to detect postpartum 2h hemorrhage (12). Postpartum recovery includes assessment of sleep, lactation, mental health, and infection. The total score of each item was 10 points, <8 was bad, and >8 was good (13).

### Statistical methods

SPSS19.0 (Shanghai Kabei Information Technology Co., Ltd.) was used for statistical analysis. The enumeration data were expressed as [n(%)], and the ratio was compared using the χ^2^ test. Measurement data were expressed as x±SD, and t-test was used for comparison between the two groups. *P*<0.05 for the difference was statistically significant.

## Results

### The pain rate of the two groups of women

There were slightly fewer cases of grade 3 pain in the observation group than in the control group, but there was no significant difference. The number of cases of grade 0 and 1 pain and the good rate of pain were significantly higher in the observation group than in the control group. The number of cases with grade 2 pain was significantly lower in the observation group than that of the control group (*P*<0.01) (Table 2).

**Table 2:** Comparison of pain rates between the two groups [n (%)]

***Group***	***Grade 0***	***Grade 1***	***Grade 2***	***Grade 3***	***Good rate of pain(%)***
Observation (n=100)	18(18.00)	69(69.00)	10(10.00)	3(3.00)	87(87.00)
Control (n=92)	6(6.52)	50(54.35)	27(29.35)	9(9.78)	56(60.87)
χ^2^	5.772	4.365	11.530	3.762	17.213
*P*	0.017	0.039	0.001	0.073	<0.001

### Comparison of the second stage of labor and the 2h postpartum hemorrhage

The second stage of labor and the 2h postpartum hemorrhage in the observation group were significantly lower than those in the control group (*P*<0.001) (Table 3).

**Table 3:** Comparison of the second stage of labor and the 2h postpartum hemorrhage in the two groups

***Group***	***second stage of labor (min)***	***2h postpartum hemorrhage (mL)***
Observation (n=100)	46.83±12.37	216.86±35.76
Control (n=92)	55.17±11.09	248.25±28.33
*t*	4.903	6.703
*P*	<0.001	<0.001

### Comparison of postpartum recovery in two groups of women

The number of maternal cases with good post-partum sleep, good lactation and mental health in the observation group was significantly higher than that in the control group (*P*<0.001). The number of infection cases in the observation group was slightly less than that of the control group, but no significant difference (Table 4).

**Table 4:** Comparison of postpartum recovery in two groups [n(%)]

***Group***	***Good sleep***	***Good lactation***	***Healthy mental***	***Infection***
Observation (n=100)	89(89.00)	92(92.00)	95(95.00)	3(3.00)
Control (n=92)	71(77.17)	73(79.35)	79(85.87)	9(9.78)
χ^2^	4.825	6.347	4.702	3.762
*P*	0.033	0.013	0.045	0.073

## Discussion

Various problems may arise from childbirth, such as pain, poor postpartum recovery, etc., so the mother needs fine care. Psychological nursing can effectively suppress negative emotions such as fear and depression, establish a positive and optimistic attitude, and reduce the incidence of postpartum depression (14); dietary guidance can help maternal nutrition, promote lactation and enhance neonatal immunity (15); behavioral care helps maternal uterine contractions, promotes blood stasis elimination, and wound healing (16); postpartum health care can prevent postpartum infections and reduce the incidence of infections; pain care can alleviate patient pain and repair trauma during childbirth as early as possible (17); breastfeeding care is conducive to the increase in breastfeeding rate, helping women learn the correct feeding posture, and reduce the discomfort caused by improper feeding (18).

This study showed that the number of cases of grade 3 pain was slightly less in the observation group than in the control group, but there was no significant difference. The number of cases of grade 0 and 1 pain and the good rate of pain were significantly higher in the observation group than in the control group, the number of cases with grade 2 pain in the observation group was significantly lower than that of the control group. This indicates that the maternal pain of fine care is lower than that of women receiving routine care, it is probably because of the fine care making the mother fully prepared before giving birth and positively facing and accepting the situation during the childbirth, cooperating with the doctor through breathing to complete the delivery and reduce the incidence of tearing and side cutting (19).

The second stage of labor and the 2h postpartum hemorrhage in the observation group were significantly lower than the control group. Due to the complexity and variability of the delivery process, it is easy for the mother to have negative emotions, thus the heart rate is accelerated, the gas exchange in the lung is insufficient, resulting in uterine hypoxia and lack of contraction. The slow expansion of the cervix leads to prolonged labor (20, 21). This process also causes changes in maternal neuroendocrine and inhibits oxytocin, which increases the incidence of postpartum hemorrhage (22). The 2h postpartum hemorrhage accounted for 75% of the bleeding volume at 24h postpartum (23), indicating that the key to preventing postpartum hemorrhage is to strengthen the care 2 hours after delivery. The number of maternal cases with good postpartum sleep, good lactation and mental health in the observation group was significantly higher than that in the control group. The number of infection cases in the observation group was slightly less than that of the control group, but no significant difference.

Early postpartum contact between the infant and the mother and sucking the nipple can stimulate the release of maternal oxytocin to reduce the postoperative pain and the incidence of complications, but also stimulate the mother to produce milk, while increasing the emotional communication between the mother and the newborn (24). Breastfeeding can promote the recovery of body and immune function (25).

## Conclusion

Prenatal fine care can reduce maternal pain rate, shorten the second stage of labor, reduce the amount of postpartum 2h bleeding, while sleep, lactation, psychological conditions are promoted, postpartum infection rate is reduced, which is conducive to maternal body recovery, worthy of clinical promotion.

## Ethical considerations

Ethical issues (Including plagiarism, informed consent, misconduct, data fabrication and/or falsification, double publication and/or submission, redundancy, etc.) have been completely observed by the authors.
